# Deadly confusion of novel psychoactive substances: fatal outcome of ADB-BUTINACA mislabeled as 3’,4’-methylenedioxy-α-pyrrolidinohexiophenone

**DOI:** 10.1007/s11419-025-00746-z

**Published:** 2025-11-27

**Authors:** Annette Zschiesche, Nadine Theofel, Stefan Braukmüller, Edwin Ehrlich, Martin Jasyk, Maximilian Methling, Michael Tsokos, Stefan Scholtis, Laura M. Huppertz, Volker Auwärter

**Affiliations:** 1 Institute of Forensic Medicine, Forensic Toxicology, Medical Center - University of Freiburg, Faculty of Medicine, University of Freiburg, Albertstr. 9, 79104 Freiburg, Germany; 2 Hermann Staudinger Graduate School, University of Freiburg, Hebelstr. 27, 79104 Freiburg, Germany; 3 Department of Forensic Toxicology, Governmental Institute of Legal Medicine and Forensic Sciences, Turmstraße 21, Berlin, Germany; 4 Institute of Organic Chemistry, University of Freiburg, 79104 Freiburg, Germany

**Keywords:** Fatal intoxication, NPS, Synthetic cannabinoid receptor agonist, Designer stimulant, Mislabeling, Drug-checking

## Abstract

**Purpose:**

A powder found at a fatality scene, labeled as the synthetic cathinone 3’,4’-methylenedioxy-α-pyrrolidinohexiophenone (MDPHP) and most likely smoked using a crack pipe, was analyzed. The powder was identified as very potent synthetic cannabinoid ADB-BUTINACA/ADB-BINACA with a high purity (> 98%). This case highlights the risks associated with mislabeled novel psychoactive substances (NPS), particularly those purchased online.

**Methods:**

The powder was analyzed using liquid chromatography-high resolution mass spectrometry (LC-HRMS), liquid chromatography-tandem mass spectrometry (LC-MS/MS), and nuclear magnetic resonance (NMR) spectroscopy. Comprehensive toxicological screening, including NPS, was performed in urine and blood. ADB-BUTINACA was quantified using a standard addition method on various post-mortem matrices: femoral and heart blood, urine, stomach content, bile fluid and liver tissue. Scalp hair was analyzed via external calibration to assess potential long-term exposure.

**Results:**

ADB-BUTINACA concentrations were 34.5 ng/mL in femoral blood, 101 ng/mL in heart blood and 3.1 ng/mL in urine. Traces of MDMB-BUTINACA were found in the powder and hair, but not in other biological matrices. ADB-BUTINACA metabolites were detected in all biological matrices. MDPHP was found at low concentrations (< 2 ng/mL) in blood and urine but not in the powder, indicating prior cathinone use. A toxicological significance score (TSS) of 3 was assigned for this monointoxication with ADB-BUTINACA.

**Conclusions:**

This case demonstrates fatal poisoning due to extremely high ADB-BUTINACA concentrations in post-mortem blood samples, emphasizing the severe risks associated with mislabeled substances. It underscores the importance of drug checking services to prevent poisonings and overdoses caused by highly potent NPS.

**Supplementary Information:**

The online version contains supplementary material available at 10.1007/s11419-025-00746-z.

## Introduction

Mislabeling of substances presents a significant public health risk, particularly in the context of so-called new psychoactive substances (NPS). This is evident in cases where cannabis products are adulterated with synthetic cannabinoids (SCs), which imitate the effects of Δ⁹-THC but often act as full agonists at the human CB_1_ receptor (hCB_1_), making them significantly more dangerous [1]. In the following studies, samples were analyzed using either liquid chromatography-tandem mass spectrometry (LC-MS/MS) or gas chromatography-mass spectrometry (GC-MS). In Switzerland, a study found that 50% of 94 ‘low-THC’ (< 1% Δ9-THC) cannabis samples contained up to three synthetic cannabinoids [2]. Similarly, Oomen et al. reported that approximately 23.6% of 1,142 products sold as cannabis (herbal material, e-liquids, and resins) across eight countries contained the potent SC MDMB-4en-PINACA [3]. Analysis of nine cannabidiol (CBD) e-liquids revealed presence of 5F-ADB (5F-MDMB-PINACA) – a highly potent SC – in four samples [4]. In a recent case in France, a person experienced severe symptoms after smoking 300 mg of a substance labeled as DMT, which actually contained the potent synthetic opioid protonitazene. The symptoms required repeated naloxone administration [5]. Such cases highlight how mislabeling of NPS products can lead to severe and unexpected intoxications, emphasizing the urgent need for awareness and stringent quality control.

Fatalities linked to synthetic cannabinoids are well documented. Substances such as 5F-Cumyl-PEGACLONE [6] and 5F-ADB (5F-MDMB-PINACA) [7–9] have been associated with deaths, as summarized in the post-mortem review by Giorgetti et al. [10]. In Munich, Groth et al. reported 98 fatalities related to synthetic cannabinoids, involving 41 different SCs – many with high toxicological significance scores (TSS) [11]. One notable case involved the SC ADB-HEXINACA, first detected in Europe in mid-2021, as well as ADB-BUTINACA and 4F-MDMB-BICA, its hydrolysis product and the hydrolysis product of MDMB-4en-PINACA [12]. According to the World Health Organization (WHO), ADB-BUTINACA has been implicated in at least six fatal and eight nonfatal poisonings [13]. An earlier report described an unintentional intoxication after vaping ADB-BUTINACA, although no concentrations were determined [14]. In another incident, a police dog died after likely inhaling ADB-BUTINACA dust [15].

**Fig. 1:**
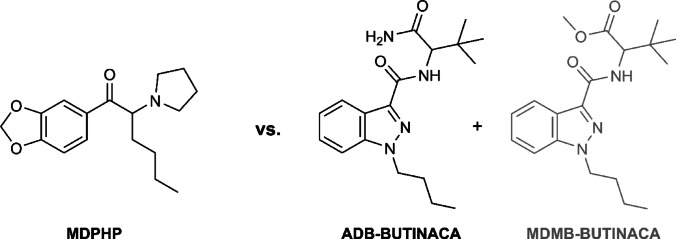
Structures of MDPHP and the actual ingredients of the powder: ADB-BUTINACA (main ingredient) and MDMB-BUTINACA (trace amounts, depicted in gray)

ADB-BUTINACA (also known as ADB-BINACA or ADMB-BINACA; IUPAC: *N*-(1-amino-3,3-dimethyl-1-oxobutan-2-yl)-1-butyl-1*H*-indazole-3-carboxamide) remains a prevalent SC in Europe [2, 12]. Its structure is shown in Fig. 1. In vitro at CB_1_, ADB-BUTINACA, measured with assays based on different assay principles, showed high potency with EC_50_ values of 0.67 nM, 6.36 nM, and 11.5 nM. The corresponding E_max_ values were 113% (CP55,940), 290% (JWH-018), and 121% (JWH-018) – reference compounds are indicated in parentheses – confirming full agonist activity. The differences in pharmacological data for ADB-BUTINACA can be explained by variations in assay conditions, reference compounds, and the specific signaling pathway utilized [16–18]. This compound, the *N*-butyl homolog of ADB-PINACA, was first reported to the EU Drugs Agency (EUDA, formerly EMCDDA) by Sweden in September 2019 [19]. It is now controlled under Germany’s Narcotic Drugs Act (BtMG, Amendment II) [20] and the New Psychoactive Substances Act (NpSG) [21]. User reports describe effects of ADB-BUTINACA such as euphoria, extreme intoxication (‘mad stoned’), warm sensations, and sedation; higher doses may cause sedation and sleepiness [13, 22]. In England, emergency department presentations following ADB-BUTINACA exposure included agitation, tachycardia, reduced consciousness, hallucinations, seizures, hypotension, and metabolic or respiratory acidosis [23]. Additional risks associated with ADB-BUTINACA include cardiac and neurological effects, as well as oxidative stress [24]. More broadly, SCs are known for their addictive potential and adverse behavioral impacts [25].

MDPHP (3,4-MDPHP also known as 3,4-MDPHP, 3’,4’-methylenedioxy-α-pyrrolidinohexiophenone, IUPAC: 1-(1,3-benzodioxol-5-yl)-2-(pyrrolidin-1-yl)hexan-1-one), a synthetic cathinone of the pyrovalerone type, acts as a potent norepinephrine-dopamine reuptake inhibitor. The structure is shown in Fig. 1. Kolaczynska et al. reported IC₅₀ values for NET, DAT, and SERT as 60 nM, 50 nM, and 9,000 nM [26]. Its street name ‘monkey dust’ is shared with the closely related cathinone MDPV (methylenedioxypyrovalerone). It was associated with severe neurological and cardiovascular symptoms, including psychomotor agitation, aggression, and cardiac disturbances [27, 28]. MDPHP has also been reported in chemsex settings, where it is used to heighten sensation and reduce inhibition [29, 30].

Drug checking services have been shown to be a valuable tool for harm reduction, but are still not available in many parts of Germany [31–34]. They usually provide analytical characterization of substances delivered by the consumers and combine information on drug composition with counseling designed to encourage consumers to reflect on their consumption behavior.

This study aimed to identify and characterize a potentially mislabeled powder found at a fatality scene. Further, ADB-BUTINACA was quantified in a comprehensive set of post mortem matrices with the standard addition method. Finally, the significance of ADB-BUTINACA as the cause of death was assessed.

## Case history

A 26-year-old man (62 kg, 172 cm, BMI 21) was found deceased in his apartment. His last known contact occurred the evening before discovery, during which he reported to experience mild cold symptoms. The decedent was a known smoker and had a medical history of bronchial asthma. His mother reported no known history of illicit drug use, and no suicide note was recovered at the scene.

**Fig. 2:**
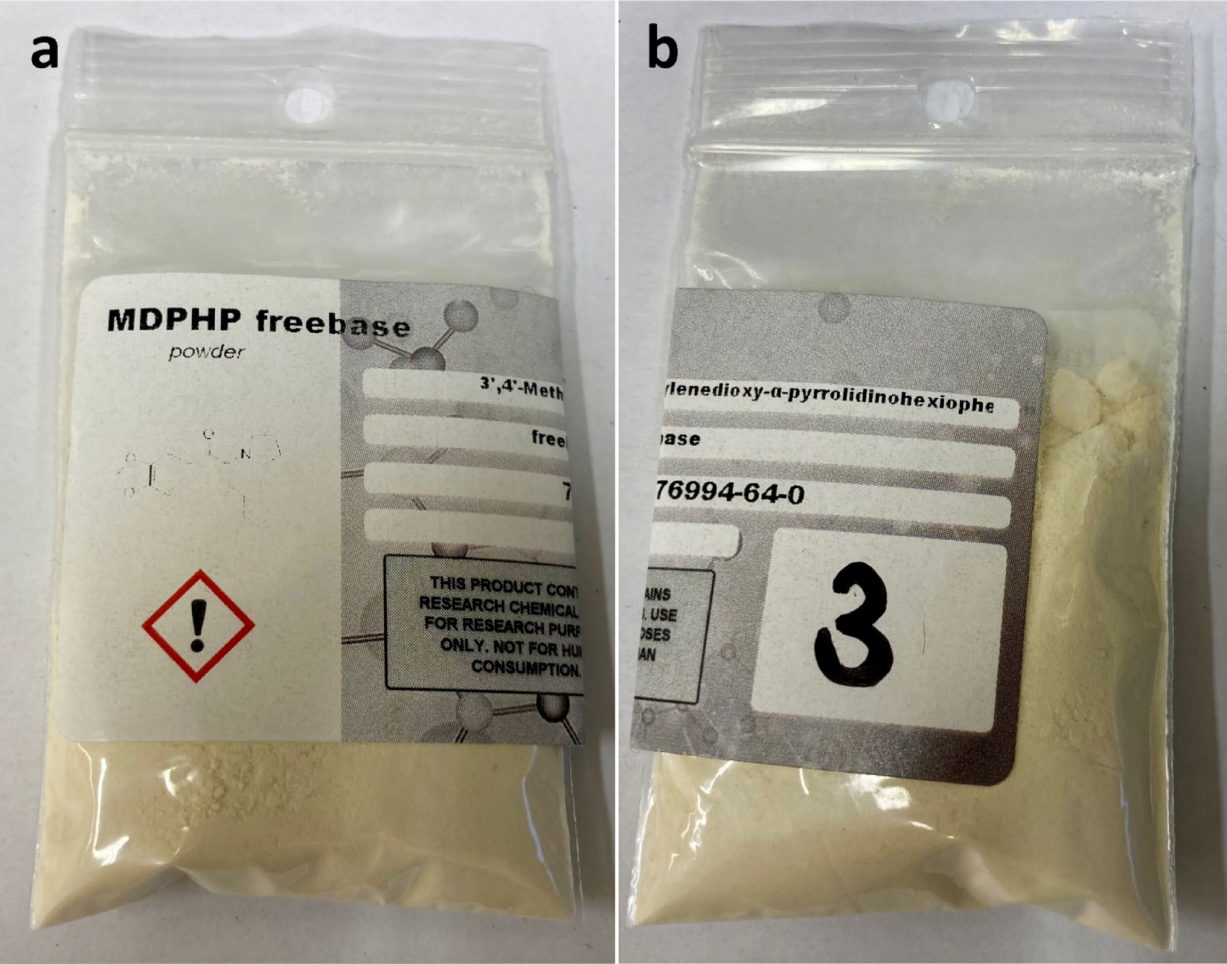
Pictures of the package with the yellowish powder labeled as ‘MDPHP freebase’: (**a**) front side, (**b**) back side

The body was discovered wearing only a ‘mankini’ and positioned partially on the bed and partially on an adjacent side table, in a twisted posture. In close proximity to the body, investigators found scissors, a cut piece of aluminium foil, and a gas burner. On a nearby table, a spoon, a crack pipe, and a plastic bag labeled ‘MDPHP freebase’ containing a yellowish powder were also secured (see Fig. 2). Additionally, a shipment from the Netherlands, including an English safety data sheet for MDPHP, was recovered in the kitchen.

The autopsy was conducted 10 days after the body was discovered and stored at 4 °C during this time.

## Materials and methods

### Reference materials and chemicals

Acetonitrile (ACN, LC-MS grade) and ammonium formate (10 M) were bought from Sigma-Aldrich (Steinheim, Germany). Deionized water was prepared using a Medica^®^ Pro deionizer from ELGA (Celle, Germany). Formic acid (p.a.) and methanol were from Carl Roth GmbH (Karlsruhe, Germany). Isopropanol (Prepsolv^®^) was obtained from Merck (Darmstadt, Germany).

Pooled human liver microsomes (pHLMs, 200 donors, 20 mg/mL protein in 250 mM sucrose) were from XenoTech (Kansas City, USA). The NADPH-regenerating solutions A (26 mM NADP^+^, 66 mM glucose-6-phosphate and 66 mM MgCl_2_ in H_2_O) and B (40 U/mL glucose-6-phosphate dehydrogenase in 5 mM sodium citrate) with a reductase activity of 0.43 µmol/min × mL and the potassium phosphate buffer 0.5 M (pH 7.4) were purchased from Corning (Amsterdam, the Netherlands). Roche Diagnostics (Mannheim, Germany) supplied the β-glucuronidase (*Escherichia coli* K12) used for conjugate cleavage.

The reference standards ADB-BUTINACA and ADB-BUTINACA *N*-3OH butyl were purchased from Chiron AS (Trondheim, Norway) whereas MDMB-BUTINACA butanoic acid metabolite (ADB-BUTINACA metabolite A6/M1), ADB-INACA and the internal standard (IS) AB-PINACA-d9 were purchased from Cayman Chemical (Ann Arbor, Michigan, USA). The reference substance MDPHP (75% purity) was obtained from the RESPONSE project in Ljubljana, Slovenia, while PCP-d5 was purchased from Lipomed (Weil am Rhein, Germany).

### Post-Mortem examination and sampling

The autopsy involved a full post-mortem examination, along with the collection of biological fluids and tissues for toxicological analysis. The collected specimens included femoral and cardiac blood, urine, gastric contents, bile fluid, liver tissue, and scalp hair (0–4.5 cm, proximal segment). Biological materials were stored at 4 °C for approximately 16 weeks in Berlin, and subsequently at − 20 °C in Freiburg prior to the analysis.

### General toxicological analysis

General unknown screening was performed using various techniques varying from LC-high resolution MS (HRMS), GC-MS, immunological screening to targeted LC-MS/MS methods and NMR spectroscopy. The following section details which matrices were examined and which methods were applied.

#### Powder sample

One milligram of the powder was dissolved in acetonitrile and diluted to 100 ng/mL. General unknown LC-HRMS screening was conducted. An Elute HPLC system coupled with an Impact II QToF instrument (Bruker Daltonik, Bremen, Germany) operated in heated electrospray ionization (HESI) positive mode using Bruker’s TargetScreener HR 4.0 was applied [35]. Both an in-house database and the HighResNPS database [36] were used. Additionally, it was analyzed for synthetic cannabinoids (SCs, described in ‘Screening of Femoral and Heart Blood’) and designer stimulants (described in ‘Screening of Urine’) using targeted LC-MS/MS methods.

To verify the identity and purity of the powder, one-dimensional ^1^H-NMR (700 MHz) and ^13^C-NMR (176 MHz) spectra were acquired, as well as selective 2D spectra including ^1^–^13^ C edited heteronuclear single quantum coherence (HSQC), ^1^H–^1^H double quantum filtered correlation spectroscopy (DQFCOSY), ^1^–^13^ C heteronuclear multiple quantum coherence (HMBC), and ^1^–^15^ N heteronuclear multiple quantum coherence (HMBC-^15^N). Measurements were performed in DMSO-d6 at 300 K using a Bruker Avance Neo 700 NMR spectrometer (Bruker BioSpin, Rheinstetten, Germany) with a BBO-Prodigy-Probehead. Chemical shifts were referenced internally to the ^1^H- and ^13^C-NMR signals of DMSO-d6.

#### Screening of urine

Urine was centrifuged (12,000×g, 3 min), and the supernatant was screened via immunoassay for opiates, benzodiazepines, amphetamines, cannabinoids, cocaine and metabolites, methadone, barbiturates and salicylate using an Indiko Plus instrument (Thermo Fisher Scientific, Dreieich, Germany). The immunoassay cut-offs were as follows: barbiturates and benzodiazepines (200 µg/L), opiates and methadone (300 µg/L), cocaine (150 µg/L), cannabinoids (50 µg/L), amphetamine (500 µg/L), and salicylate (100 µg/mL).

Subsequent systematic toxicological analysis was performed using a Finnigan Trace GC 2000 system equipped with a Programmable Temperature Vaporizer injector (Thermo Quest, Manchester, UK). A Zebron ZB-5MSi capillary column (30 m × 0.25 mm × 0.25 μm; Phenomenex, Torrance, USA) was used.

For GC-MS toxicological screening, the urine sample was adjusted to pH 5–7 using 2 mL of 0.1 M phosphate buffer (pH 6, adjusted with 0.1 M HCl). Solid-phase extraction (SPE) was performed using Bond Elut LRC cartridges (130 mg sorbent, Agilent Technologies, USA). Cartridges were preconditioned with 2 mL methanol and 2 mL phosphate buffer. After sample application (2 mL), the cartridges were sequentially washed with 1 mL 1 M acetic acid and 5 mL methanol to remove interfering substances. Alkaline analytes were subsequently eluted with 2 mL of a solvent mixture of methylene dichloride, 2-propanol, and 25% ammonia (40:10:1, v/v).

The eluate was reduced by one-third at 40 °C by evaporation under a nitrogen stream. Then, 50 µL of 2-propanol/0.1 M HCl was added, evaporated under nitrogen, and reconstituted in 100 µL methanol. Two aliquots (50 µL each) were prepared: Sample 1 was analyzed directly via GC-MS; whereas Sample 2 was derivatized with 50 µL pyridine and 100 µL acetic anhydride at 80 °C for 30 min, then evaporated and reconstituted in 100 µL methanol for GC-MS analysis.

For acidic analytes, cartridges were washed with 1 mL phosphate buffer/methanol (4:1, *v/v*), 1 mL 1 M acetic acid, and 1 mL hexane. Elution was performed with 2 mL acetone, followed by nitrogen drying and reconstitution in 100 µL methanol. An aliquot (50 µL) was derivatized using 20 µL trimethylsulfonium hydroxide in 50 µL methanol at 80 °C for 30 min before GC-MS analysis (Sample 3).

Urine was analyzed using forensically accredited and validated methods including LC-HR-MS screening and LC-MS/MS-methods, updated to include newly occurring SCs (metabolites of 87 SCs), designer stimulants (173 analytes), hallucinogens (73 analytes), designer benzodiazepines (109 analytes) and opioids (125 analytes) [28, 37, 38] covering all relevant compounds currently available from online stores active in Europe.

SC parent substances are mostly not detectable in urine [12, 39]. For the detection of metabolites of synthetic cannabinoids, urine was hydrolyzed enzymatically using β-glucuronidase and extracted with acetonitrile containing deuterated internal standards using a salting-out assisted liquid-liquid with ammonium formate (10 M) extraction protocol [38, 40]. After centrifugation and evaporation, residues were reconstituted in mobile phase and analyzed for phase I SC metabolites.

For MDPHP detection, Multiple Reaction Monitoring (MRM) transitions of *m/z* 290.2 → 135.0 and *m/z* 290.2 → 219.1 were used; the internal standard was PCP-d5 (*m/z* 239.1→164.1). The LC-MS/MS method followed Grapp et al. [28] with a 1–50 ng/mL calibration range. Urine (0.5 mL) was extracted using Chromabond^®^ drug cartridges (Macherey-Nagel, Düren, Germany). To prevent amine volatilization, 100 µL of a hydrochloric acid-isopropanol solution (3:1, *v/v*) was added prior to evaporation. Separation was achieved on a Kinetex^®^ Biphenyl column (Phenomenex, Aschaffenburg, Germany) using a 24 min gradient.

Urinary creatinine was measured using a cobas^®^ 6000 analyzer with c501 module and Creatinine Jaffé Gen.2 reagent (Roche Diagnostics GmbH, Mannheim, Germany).

#### Screening of femoral and heart blood

Blood samples were analyzed using the aforementioned LC-HRMS and the targeted LC-MS/MS-method for SCs (123 parent substances and respective hydrolysis metabolites). The methods used for designer stimulants and hallucinogens were the same as described in the section ‘Screening of urine’ with sample volumes of 0.5 mL.

For SC detection, 200 µL of blood was fortified with internal standards and extracted with ammonium formate (10 M) and 1 mL acetonitrile. After evaporation, samples were reconstituted in mobile phase and analyzed using LC-MS/MS with an Ultimate 3000RS UHPLC (Dionex, Sunnyvale, USA) coupled to a QTRAP^®^ 6500 system (SCIEX, Darmstadt, Germany) in positive ESI mode. Chromatography was conducted on a Kinetex^®^ C18 column (2.6 μm, 100 Å, 100 × 2.1 mm; Phenomenex, Aschaffenburg, Germany) following the gradient described by Huppertz et al. [41]. A scheduled MRM (sMRM) method was used with two transitions per analyte and one for the internal standard. Parameters (DP, EP, CE, CXP) were optimized. Calibration ranged from 0.1 to 10 ng/mL. For ADB-BUTINACA, the transitions *m/z* 331.2 → 201.1 and *m/z* 331.2 → 286.2 were used. AB-PINACA-d9 (*m/z* 340.3 → 224.2) served as internal standard.

#### Scalp hair samples

Scalp hair (0–4.5 cm segment) was analyzed for SCs (same analytes as for blood) following the protocols of Franz et al. [42] and designer stimulants (same analytes as for blood and urine). The hair sample (106 mg) was decontaminated with deionized water, acetone, and petroleum ether, and then dried. An aliquot (20.4 mg) was cut into 1–2 mm pieces, spiked with internal standards, and extracted with 1.5 mL methanol under ultrasonication (3 h). The extract was evaporated and reconstituted in mobile phase. Calibration ranged from 5 to 100 pg/mg for SCs and 10–200 pg/mg for stimulants.

Aliquots (0.25 mL) of each washing solution were combined, and internal standards added. The solution was evaporated under nitrogen at 40 °C, reconstituted in eluent, and analyzed by LC-MS/MS with the same methods applied for the hair sample.

### Standard addition method (SAM)

The standard addition method (SAM) was used to correct for matrix effects common in post-mortem specimens [43].

Femoral and heart blood, urine, bile, and homogenized stomach contents were diluted with phosphate buffer (pH 6) before SAM application (dilution factors in Table 1) in order to operate in the linear range of the mass spectrometer.

**Table 1:** Results for scalp hair and the corresponding combined washing solutions

	c(ADB-BUTINACA) [pg/mg]	c(MDPHP) [pg/mg]
Scalp hair sample (20.4 mg)	250	220
Combined washing solutions	390	530
Wash-to-hair-ratio	1.5	2.4

Liver tissue (0.5 g) was minced with clean surgical scissors, homogenized in a 1.5 mL tube with ceramic beads and 1 mL phosphate buffer using a BeadBug homogenizer (Süd-Laborbedarf GmbH, Gauting, Germany), then centrifuged (2,898×g, 10 min). The supernatant was diluted 1:5 with phosphate buffer (pH 7.4) as detailed in Table 2.

Each matrix was processed using 100 µL of the diluted sample, with a six-point calibration curve (0, 1, 2.5, 5, 7.5, 10 ng/mL) by spiking an acetonitrile solution containing 100 ng/mL of ADB-BUTINACA. Samples were fortified with 1 mL acetonitrile containing AB-PINACA-d9 (0.5 ng/mL) and 100 µL ammonium formate. Following processing, samples were shaken for 10 min, centrifuged (2,898×g, 10 min) and the organic layer was evaporated under a stream, reconstituted in 100 µL eluent (80:20, *v/v*), and analyzed by LC-MS/MS. Each SAM was performed in triplicate for every matrix.

### Instrumental conditions used for SAM

Analysis was conducted using an Ultimate 3000RS UHPLC system (Dionex, Sunnyvale, USA), coupled with a QTRAP^®^ 6500 triple quadrupole-linear ion trap mass spectrometer (SCIEX, Darmstadt, Germany) operating in positive electrospray ionization (ESI^+^) mode.

Chromatographic separation was achieved using a Kinetex^®^ C18 column (2.6 μm, 100 Å, 100 × 2.1 mm; Phenomenex, Aschaffenburg, Germany) with an injection volume of 10 µL and a total runtime of 8.25 min. The following source parameters were used: curtain gas, 25 psi; collision gas, “high”; ion spray voltage, 5,500 V; source temperature, 600 °C; nebulizer gas, 30 psi; and heater gas, 20 psi. Nitrogen served as collision and curtain gas. Retention time, transitions and ion source parameters of ADB-BUTINACA and AB-PINACA-d9 can be found in Table S1.

Mobile phase A consisted of 1% acetonitrile, 0.1% formic acid, and 2 mM ammonium formate in water, while mobile phase B included 0.1% formic acid and 2 mM ammonium formate in acetonitrile. Mobile phases were freshly prepared prior to analysis. The gradient elution program was as follows: 0.00–0.50 min: 25% B at 0.45 mL/min (increased to 0.50 mL/min); 6.50 min: gradient raised to 70% B at 0.50 mL/min; 6.80 min: increased to 90% B at 0.55 mL/min; 7.75 min: maintained at 0.60 mL/min; 7.85 min: return to 25% B at 0.45 mL/min; 8.25 min: end of run.

Data acquisition and processing were carried out using Analyst version 1.6.3 (Sciex, Darmstadt, Germany), and subsequent data handling was performed using Microsoft Excel 2007 (Microsoft Corporation, Redmond, WA, USA).

### Metabolites of ADB-BUTINACA

An in vitro pooled human liver microsome (pHLM) assay was performed to generate a reference for assessment of retention times of the phase I metabolites of ADB-BUTINACA, following established methods [40]. A final concentration of 10 µg/mL of ADB-BUTINACA (dissolved in acetonitrile) was used for incubation with pHLMs, phosphate buffer, and the necessary enzyme cofactors at 37 °C for 30 min. The reaction was terminated by the addition of ice-cold acetonitrile and 10 M ammonium formate. Controls included a substance blank (without pHLMs) and a negative control (without ADB-BUTINACA). The samples were centrifuged (10 min at 16,550×g). Afterwards, 5 µL of the organic layer were dried by a gentle steam of nitrogen and reconstituted in 50 µL of eluent A/B (*v/v* 80/20).

For further control of retention times, covering metabolites not built in vitro, two serum and urine samples, previously found positive for ADB-BUTINACA and several of its metabolites during routine forensic casework, were processed in the same way as post-mortem blood and urine.

To evaluate metabolite profiles in various post-mortem matrices, 100 µL of each undiluted biological fluid (supernatant in the case of liver tissue) was analyzed. For matrices where phase I metabolites are likely to undergo glucuronidation (e.g., urine, bile, and homogenized liver), samples were additionally subjected to enzymatic hydrolysis prior to analysis.

### Instrumental conditions used for metabolite detection

For metabolite detection, the same LC-MS/MS method as for SAM was used, but metabolites of ADB-BUTINACA and MDMB-BUTINACA were added into the MRM method. For ADB-BUTINACA *N*-3OH butyl, ADB-BUTINACA metabolite A6/M1 and ADB-INACA, reference standards were used. Other published main metabolites of ADB-BUTINACA were included by matching retention times, fragmentation patterns based on an ADB-BUTINACA pHLM incubation and serum and urine samples found positive for ADB-BUTINACA. The respective retention times, MRM transitions, and ion source parameters are listed in Table S1.

## Results

### Autopsy findings

Post-mortem examination revealed pronounced livor mortis, with *vibices* observed in the head and shoulder regions. No signs of external or internal trauma were detected. The lungs exhibited marked pulmonary edema, with weights of 620 g (right) and 580 g (left), both exceeding typical reference ranges. The brain weighed 1,535 g, displaying no macroscopic signs of trauma, although the elevated weight may indicate mild cerebral edema. The remaining organ weights were as follows: heart, 350 g; spleen, 220 g; liver, 1,420 g; right kidney, 110 g; and left kidney, 150 g. The stomach contained 30 mL of fluid and undigested vegetable material. No natural cause of death was identified macroscopically.

### Analytical findings

#### General toxicological screenings

##### Powder sample

The seized powder sample labeled as MDPHP was identified via multidimensional NMR spectroscopy as ADB-BUTINACA, with a purity exceeding 98%. Trace amounts of MDMB-BUTINACA (also known as MDMB-BINACA), the analog of ADB-BUTINACA with a methyl ester instead of an amide moiety, was also detected (LC-MS/MS). Neither the LC-HRMS screening nor the targeted LC-MS/MS method analysis for designer stimulants confirmed the presence of the initially suspected MDPHP or any other synthetic cathinone.

#### Screening of urine, femoral and heart blood

Initial immunochemical screening of urine yielded negative results. Beyond ADB-BUTINACA and trace amounts of MDPHP (LC-HRMS, LC-MS/MS), post-mortem analysis of urine and blood revealed only ibuprofen, caffeine, nicotine, and their respective metabolites (LC-HRMS, GC-MS). Urinary creatinine was 35 mg/dL. Using LC-MS/MS, MDMB-BUTINACA was also detected in the blood samples at low, non-quantifiable levels.

MDPHP was detected with concentrations of 0.3 ng/mL (extrapolated) in urine, 1.5 ng/mL in heart blood, and 0.2 ng/mL (extrapolated) in femoral blood, LOD (0.3 ng/mL).

#### Scalp hair samples

Analysis results of the black dyed scalp hair sample for synthetic cannabinoids, designer stimulants and hallucinogens (0–4.5 cm, proximal segment; 20.4 mg analyzed) are presented in Table 2. Since only a fraction of the combined wash solution was analyzed, the concentration of analytes in the wash solution was recalculated based on the total hair amount of hair, initially washed. No synthetic cannabinoids, designer stimulants, or hallucinogens were detected, except for ADB-BUTINACA, MDPHP and trace amounts of MDMB-BUTINACA, which were present but not quantified.

**Table 2:** Concentrations of ADB-BUTINACA in post-mortem matrices using SAM in triplicates. The linear regression equation and its correlation coefficient were calculated using the mean values

Matrix	Concentration [ng/mL]	Dilution factor	Equation	Correlation coefficient (*R*²)
Femoral blood	34.5 ± 4.9	20	y = 0.2661x + 0.4591	0.9945
Heart blood	101 ± 13.9	50	y = 0.2664x + 0.5380	0.9968
Urine	3.1 ± 0.5	4	y = 0.2349x + 0.3765	0.9980
Liver tissue	3.1 ± 1.5*	5	y = 0.2390x + 0.0666	0.9995
Bile fluid	34.3 ± 2.6	20	y = 0.2489x + 0.4274	0.9994
Stomach content	146 ± 5.0 (absolute: 4.38 ± 0.15 mg)	50	y = 0.2401x + 0.6994	0.9983

* in ng/g

### Standard addition method

The standard addition method (SAM) was employed for the quantification of ADB-BUTINACA in various post-mortem matrices, demonstrating excellent linearity (R² > 0.99) across all matrices. Calibration curve parameters – including calculated x-intercepts (representing concentration in ng/mL), regression equations, goodness-of-fit metrics, and dilution factors – are provided in Table 2.

### Metabolites of ADB-BUTINACA

**Fig. 3:**
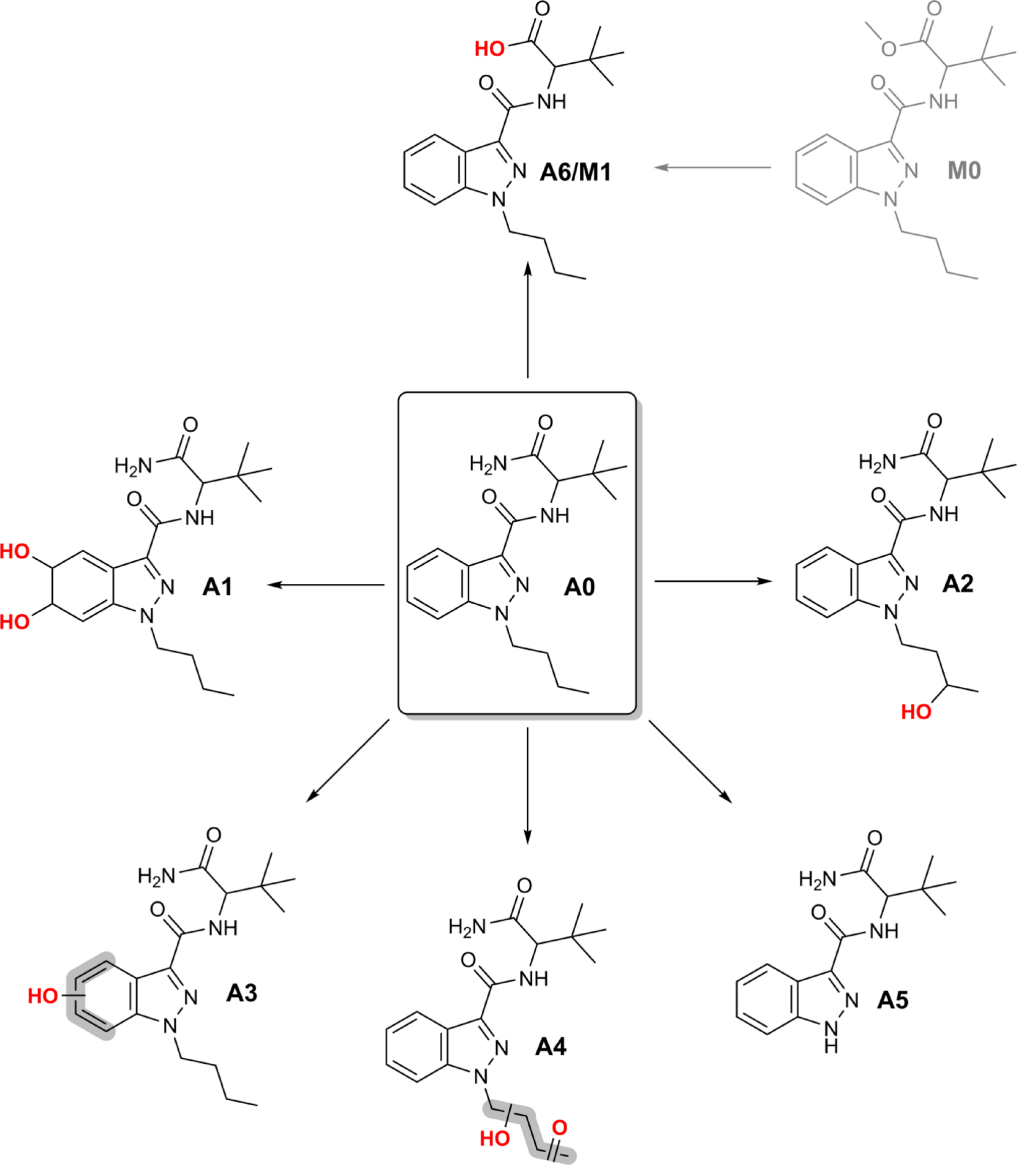
Phase I metabolites of ADB-BUTINACA (A0) detected in the different post-mortem matrices. The structure of MDMB-BUTINACA (M0) is depicted in gray to illustrate that only traces were present in the powder

All analyzed post-mortem samples were screened for previously reported phase I metabolites of ADB-BUTINACA. The chemical structures of the investigated metabolites are illustrated in Fig. 3.

All of the main phase I metabolites of ADB-BUTINACA searched for can be found in Table 3. The retention time of the metabolites were confirmed with the pHLM assay and two routine serum and urine samples from the forensic toxicological routine casework. None of the specific metabolites of MDMB-BUTINACA searched for could be found.

**Table 3:** Phase I metabolites of ADB-BUTINACA (A0) and MDMB-BUTINACA (M0) found in the post-mortem samples

Matrix	A0	A1	A2	A3	A4	A5	A6/M1	M0
Femoral blood	■	■	■	■	□	■	■	□
Heart blood	■	■	■	■	■	■	■	□
Urine	■	■	■	■	□	□	■	□
Liver	■	■	■	■	■	■	■	□
Bile fluid	■	■	■	■	□	■	■	□
Stomach content	■	■	■	■	■	■	■	□
Scalp hair	■	□	□	□	□	■	■	■
Powder	■	□	□	□	□	□	□	■

■: detected, □: not detected

The parent compound (A0) as well as the hydrolysis metabolite (A6/M1) was detected in all examined biological matrices, including blood, urine, bile, and organ homogenates. The metabolite A6/M1 may result from either amide hydrolysis of ADB-BUTINACA or hydrolysis of the methyl ester moiety of MDMB-BUTINACA. However, no specific biomarkers indicative of MDMB-BUTINACA – such as a monohydroxylated metabolites or a dihydrodiol derivative – were identified in any of the analyzed matrices.

Monohydroxylated metabolites (A2 and A3) and the dihydrodiol metabolite (A1) were consistently detected in all investigated biological matrices, with the exception of scalp hair. Conversely, MDMB-BUTINACA (M0) was only detected in scalp hair, and absent from all other biological samples.

## Discussion

### Interpretation of ADB-BUTINACA concentrations

ADB-BUTINACA is not a new synthetic cannabinoid (SC), but it has remained consistently prevalent since its initial detection on the drug market in 2019. Despite its widespread occurrence, only a few fatal cases have been documented to date [13]. Only one study reported a post-mortem blood concentration of 8.1 ng/mL in a police dog following inhalation of dust containing ADB-BUTINACA [15].

In emergency departments in the United Kingdom, blood or serum concentrations of ADB-BUTINACA in non-fatal cases ranged from 0.18 to 21.1 ng/mL. Reported routes of administration included smoking of herbal blends such as ‘Spice’, injection or smoking of contaminated heroin with or without crack cocaine, and ingestion of edibles [23].

In the monointoxication case presented here, femoral and cardiac blood concentrations of 34.5 ng/mL and 101 ng/mL, respectively, are notably high and well above previously reported clinical or fatal cases. Although, the relatively long PMI of 10 days should be taken into consideration, the storage conditions of the samples are also important. The samples were initially preserved at 4 °C in plastic containers for approximately 16 weeks and then transferred to glass containers at -20 °C after shipment as SCs are known to adsorb to lipophilic surfaces such as those found in polypropylene [44]. Consequently, it cannot be assumed that the stability of ADB-BUTINACA was maintained, and degradation processes and loss due to adsorption may have occurred. As a result, it is highly probable that the actual lethal concentration was even higher than the measured values. The police dog’s blood level of 8.1 ng/mL – already considered high in relation to our in-house generated, unpublished quantitative ADB-BUTINACA concentration data for blood and serum routine samples – further supports the significance of the concentrations observed in our case. It is important to note that canines tend to be more sensitive to cannabinoids, likely due to a higher density of CB_1_ receptors in the brain [15, 45].

A potential explanation for the unusually high concentrations lies in the misidentification of the drug as MDPHP. Reported doses for MDPHP range from 5 to 40 mg depending on the route of uptake, with 5–10 mg typical for smoking [30, 46]. In contrast, typical ADB-BUTINACA doses are approximately 1 mg. Users have reported vape liquids prepared at 0.5–5 mg/mL in propylene glycol [22, 47]. If the deceased intended to consume MDPHP and unknowingly smoked ADB-BUTINACA – possibly using a crack pipe – he may have ingested a significantly larger amount than a standard dose. In the absence of prior SC use or tolerance, the resulting exposure could have led to fatal intoxication. Tolerance development to SCs has been demonstrated in repeated-use studies [48].

ADB-BUTINACA was detected in scalp hair, probably due to external contamination, preventing assessment of prior exposure. A more detailed interpretation is provided in the section ‘Hair analysis’.

### Post-Mortem redistribution and tissue distribution

The cardiac-to-peripheral blood (C/P) ratio in this case was approximately 3, indicating post-mortem redistribution (PMR). The body was stored at 4 °C for roughly nine days before autopsy. For comparison, C/P ratios for 5F-ADB have been reported to range from 0.1 to 70, with a median of 1.7 [49].

ADB-BUTINACA has a predicted log P (octanol-water partition coefficient) of 2.76, while the more lipophilic analog ADB-HEXINACA has a log P of about 3.60 (ChemDraw Professional, ver. 23). More lipophilic SCs demonstrate higher plasma protein binding; for instance, ADB-BUTINACA binds at 90.8 ± 3.72%, while ADB-HEXINACA binds at 99.7 ± 0.08% [50]. Lipophilic SCs also accumulate in adipose tissue, contributing to redistribution processes [51].

No published quantitative data for ADB-BUTINACA in urine exist. Although it has been detected in post-mortem urine [52], the low intensity is likely due to rapid metabolism and elimination. Kronstrand et al. were unable to detect ADB-BUTINACA as a parent compound in urine [18].

The stomach content showed a very high ADB-BUTINACA concentration of 146 ng/mL (total of 4.38 mg in 30 mL), likely due to recent smoking and possible swallowed drug residues from the inhalation process. Lung tissue was unavailable in this case; however, high pulmonary concentrations would be expected shortly after smoking [10]. For comparison, the dog case revealed 6.4 pg/mg in lung tissue [15].

Given the smoking route, a first-pass effect was largely avoided, explaining the higher blood concentrations relative to liver levels. Liver concentrations were 3.1 pg/mg in this case, similar to the 1.8 pg/mg observed in the dog case, but with a much higher blood-to-liver ratio [15].

Only limited quantitative data on SC concentrations in bile exist. In this case, bile and femoral blood concentrations were nearly identical, which is atypical, as bile usually contains higher SC levels [53]. This may support the hypothesis of a rapid death.

Standard deviations of ADB-BUTINACA concentrations via standard addition in triplicates were lowest in stomach contents and bile (8% and 3%, respectively), while femoral, cardiac blood, and urine showed 14%, 12%, and 17%. Liver concentrations showed the largest variability (51%), likely related to heterogeneous distribution in the tissue.

### Toxicological significance score

The Toxicological Significance Score (TSS) in this case was assigned with the highest value of 3, based on Elliot et al.‘s criteria. This reflects a high probability that ADB-BUTINACA was the cause of death, given the autopsy findings, the absence of other contributing substances, the high potency and the elevated post-mortem blood concentrations. The TSS evaluates the role of novel psychoactive substances (NPS) in fatal cases and ranges from 1 (alternative cause of death) to 3 (likely or primary cause of death) [54].

### MDPHP concentrations

MDPHP concentrations determined via external calibration were below 2 ng/mL in heart blood, femoral blood, and urine – levels that are far below those reported in fatal intoxications. For instance, Croce et al. reported concentrations as high as 1,640 ng/mL in peripheral blood and over 12,000 ng/mL in urine [55]. Other fatal and non-fatal cases have shown wide concentration ranges, up to 4,800 ng/mL in blood and up to over 23,000 ng/mL in urine [56–59]. The low MDPHP levels found in blood and urine in this case further support that MDPHP did not contribute to the fatal outcome.

### Hair analysis

Only ADB-BUTINACA and traces of MDMB-BUTINACA were found in the hair. ADB-BUTINACA concentrations reached 250 pg/mg, which is very high and suggests recent or heavy exposure [42].

In a lethal case after MDPHP consumption reported by Croce et al., hair segments 0–1.5 cm and 1.5–3.0 cm showed concentrations of 152 ng/mg and 451 ng/mg, respectively [55]. The observed concentrations are similar to the 226 pg/mg measured in the 0–4.5 cm segment of hair in the current case, which strongly supports previous MDPHP exposure, most likely due to handling the substance in powder form. In other cases, MDPHP concentrations in users scalp hair ranged from 6 to 1,000 pg/mg [60]. However, the wash-to-hair ratios of approximately 1.5 for ADB-BUTINACA and 2.4 for MDPHP exceed the contamination threshold of 0.5 [61, 62], indicating external contamination as a major source, likely from powder handling or sidestream smoke [63, 64]. Blood or other body fluids may have also contaminated the hair with ADB-BUTINACA during autopsy. Consequently, no clear conclusions about long-term use can be drawn based on the hair results. Additionally, it should be noted that the hair was dyed. The altered hair structure could have reduced the amount of drugs in the scalp hair. This has been proven for other drugs previously [65].

### ADB-BUTINACA metabolites

ADB-BUTINACA is metabolized primarily by the CYP450 isoforms CYP2C19, CYP3A4, and CYP3A5 [66]. Dihydrodiol (A1) and monohydroxylated metabolites (A2 and A3) were detected in liver and blood, consistent with previous findings [15, 52]. The unspecific *N*-debutylated metabolite A5 (ADB-INACA) was found in nearly all matrices to a very low extent, though not in urine in this case. Metabolite A6/M1 can be formed during both metabolism and pyrolysis, as shown for structurally related SCs before [63], and was found in all matrices, except the powder sample. A4, an *N*-butanoic acid derivative, already described [66, 67] was detected only in liver, heart blood, and stomach contents. Its limited distribution supports the hypothesis of a rapid fatal outcome preventing extensive distribution of oxidative metabolites. Major metabolites as the dihydrodiol were present at low levels, especially in urine, further supporting the hypothesis that death occurred shortly after intake. Groth et al. observed declining parent compound concentrations with longer post-mortem intervals, consistent with rapid SC metabolism [11]. In rats, ADB-BUTINACA exhibits fast absorption and metabolism after oral uptake (t_max_ = 2.8 h, t_½_ = 4.4 h) [68].

## Prevalence of ADB-BUTINACA in post-mortem cases in Freiburg

At the Institute of Forensic Medicine in Freiburg, routine screening revealed that ADB-BUTINACA, along with MDMB-4en-PINACA, was among the most frequently detected SCs, including clinical, forensic and – to a relatively small extent – post-mortem samples. Since its first detection in August 2019, ADB-BUTINACA has been found in 29 post-mortem femoral or cardiac blood samples, with a peak of 14 in 2023. By the end of 2024, ADB-BUTINACA accounted for 50.9% of SC-positive post-mortem cases in Freiburg, followed by MDMB-4en-PINACA and its hydrolysis product (28.1%).

Between July 2021 and December 2022, Giorgetti et al. found ADB-BUTINACA in 69.4% of SC positive urine and 44.8% of SC positive blood/serum samples [12].

## Conclusions

In the case presented here, a fatal monointoxication with the highly potent and prevalent SC ADB-BUTINACA is described (Toxicological Significance Score, TSS = 3). Based on the circumstances of discovery, the deceased probably intended to smoke the synthetic cathinone MDPHP – likely in a chemsex context, as suggested by wearing a ’mankini‘ – using a crack pipe. Due to mislabeling of the powdered substance – which contained ADB-BUTINACA instead of MDPHP – unexpectedly high concentrations of ADB-BUTINACA were detected in blood samples, supporting a rapid occurrence of death. This conclusion is further supported by the relatively low signal intensities of ADB-BUTINACA metabolites across different matrices. While MDPHP was likely consumed previously, as confirmed by relatively high MDPHP hair concentrations, it does not seem that this drug contributed to death due to its low levels in urine and post-mortem blood.

This case highlights the urgent need to expand the availability of drug checking services, particularly for buyers from online sources, in order to verify both the identity and purity of the substances received. Had the powder been tested through a drug checking service, the death could potentially have been prevented. Expanding access to drug checking is essential to reduce the risks associated with mislabeled and adulterated substances, and to decrease the incidence of accidental poisoning and overdose associated with unregulated (online) drug markets.

To our knowledge, this is the first documented instance in which a synthetic cannabinoid sold as a designer stimulant has resulted in death. To date, there is only one published case – involving a police dog that died after inhaling dust containing ADB-BUTINACA – that provides quantitative post-mortem findings for this substance in biological matrix.

## Supplementary Information

Below is the link to the electronic supplementary material.

Supplementary Material 1
