# Supplementary material for: Deadly confusion of novel psychoactive substances: fatal outcome of ADB-BUTINACA mislabeled as 3’,4’-methylenedioxy-α-pyrrolidinohexiophenone
Source: Forensic Toxicol. 2025 Nov 27;44(1):257–70. doi: 10.1007/s11419-025-00746-z (PMC12858572; doi:10.1007/s11419-025-00746-z)
Supplement: Supplementary file 1 — Supplementary Material 1 [file 11419_2025_746_MOESM1_ESM.docx]

**Deadly confusion of novel psychoactive substances: fatal outcome of ADB-BUTINACA mislabeled as 3',4'-methylenedioxy-α-pyrrolidinohexiophenone**

Annette Zschiesche^1,2^, Nadine Theofel^3^, Stefan Braukmüller^4^, Edwin Ehrlich^3^, Martin Jasyk^3^, Maximilian Methling^3^, Michael Tsokos^3^, Stefan Scholtis^3^, Laura M. Huppertz^1^, Volker Auwärter^1*^

^1^Institute of Forensic Medicine, Forensic Toxicology, Medical Center – University of Freiburg, Faculty of Medicine, University of Freiburg, Albertstr. 9, Freiburg 79104, Germany

^2^Hermann Staudinger Graduate School, University of Freiburg, Hebelstr. 27, Freiburg 79104, Germany

^3^Department of Forensic Toxicology, Governmental Institute of Legal Medicine and Forensic Sciences, Turmstraße 21, Berlin, Germany

^4^Institute of Organic Chemistry, University of Freiburg, Freiburg 79104, Germany

*Corresponding author: Email [volker.auwaerter@uniklinik-freiburg.de](mailto:volker.auwaerter@uniklinik-freiburg.de)

Journal name: Forensic Toxicology

**Table S1** Transitions and mass spectrometric parameters of the Multiple Reaction Monitoring (MRM) for detecting ADB-BUTINACA, some of its phase I metabolites, MDMB-BUTINACA and two specific metabolites of MDMB-BUTINACA (unscheduled, gray). DP: declustering potential, EP: entrance potential, CE: collision energy and CXP: cell exit potential

| **Analyte** | **t_R_ [min]** | **Q1 [Da]** | **Q3 [Da]** | **DP [V]** | **EP [V]** | **CE [V]** | **CXP [V]** |
| --- | --- | --- | --- | --- | --- | --- | --- |
| **A0**  (ADB-BUTINACA) | 4.44 | 331.2 | 201.1 | 55 | 10 | 35 | 15 |
|  |  |  | 286.2 | 55 | 10 | 21 | 15 |
|  |  |  | 145.0 | 55 | 10 | 55 | 13 |
| d9-AB-PINACA | 4.45 | 340.27 | 224.17 | 85 | 10 | 34 | 15 |
| **A1**  (dihydrodiol) | 1.53 | 365.2 | 235.1 | 60 | 10 | 35 | 16 |
|  |  |  | 320.2 | 60 | 10 | 18 | 20 |
|  |  |  | 179.0 | 60 | 10 | 54 | 14 |
| **A2**  (*N*-3OH-butyl) | 2.27 | 347.2 | 217.1 | 50 | 10 | 35 | 17 |
|  |  |  | 302.2 | 50 | 10 | 18 | 18 |
|  |  |  | 145.0 | 50 | 10 | 57 | 13 |
| **A3**  (monoOH indazole core) | 3.13 | 347.2 | 217.1 | 50 | 10 | 35 | 17 |
|  |  |  | 302.2 | 50 | 10 | 18 | 18 |
|  |  |  | 161.0 | 50 | 10 | 53 | 14 |
| **A4**  (*N*-butanoic acid or *N*-hydroxyketone) | 2.18 | 361.2 | 316.2 | 53 | 10 | 25 | 18 |
|  |  |  | 231.1 | 53 | 10 | 38 | 14 |
|  |  |  | 145.0 | 53 | 10 | 52 | 10 |
| **A5**  (ADB-INACA) | 1.80 | 275.2 | 230.1 | 45 | 10 | 24 | 15 |
|  |  |  | 145.0 | 45 | 10 | 47 | 11 |
| **A6/M1**  (hydrolysis product) | 5.14 | 332.2 | 201.1 | 50 | 10 | 35 | 17 |
|  |  |  | 145.0 | 50 | 10 | 57 | 14 |
|  |  |  | 286.2 | 50 | 10 | 27 | 20 |
| **M0**  (MDMB-BUTINACA) | 6.65 | 346.2 | 201.1 | 86 | 10 | 29 | 6 |
|  |  |  | 286.2 | 86 | 10 | 21 | 18 |
|  |  |  | 145.0 | 86 | 10 | 57 | 16 |
| MDMB-BUTINACA MonoOH | - | 362.2 | 217.1 | 70 | 10 | 37 | 15 |
|  |  |  | 302.2 | 70 | 10 | 23 | 17 |
|  |  |  | 201.1 | 70 | 10 | 39 | 14 |
|  |  |  | 161.0 | 70 | 10 | 55 | 12 |
|  |  |  | 145.0 | 70 | 10 | 58 | 11 |
| MDMB-BUTINACA dihydrodiol | - | 380.2 | 235.1 | 70 | 10 | 38 | 15 |
|  |  |  | 320.2 | 70 | 10 | 23 | 18 |
|  |  |  | 179.0 | 70 | 10 | 56 | 11 |
